# Comprehensive proteomic quantification of bladder stone progression in a cystinuric mouse model using data-independent acquisitions

**DOI:** 10.1371/journal.pone.0250137

**Published:** 2022-06-30

**Authors:** Jacob Rose, Nathan Basisty, Tiffany Zee, Cameron Wehrfritz, Neelanjan Bose, Pierre-Yves Desprez, Pankaj Kapahi, Marshall Stoller, Birgit Schilling

**Affiliations:** 1 Buck Institute for Research on Aging, Novato, CA, United States of America; 2 University of California San Francisco, San Francisco, CA, United States of America; H Lee Moffitt Cancer Center and Research Institute, UNITED STATES

## Abstract

Cystinuria is one of various disorders that cause biomineralization in the urinary system, including bladder stone formation in humans. It is most prevalent in children and adolescents and more aggressive in males. There is no cure, and only limited disease management techniques help to solubilize the stones. Recurrence, even after treatment, occurs frequently. Other than a buildup of cystine, little is known about factors involved in the formation, expansion, and recurrence of these stones. This study sought to define the growth of bladder stones, guided by micro-computed tomography imaging, and to profile dynamic stone proteome changes in a cystinuria mouse model. After bladder stones developed *in vivo*, they were harvested and separated into four developmental stages (sand, small, medium and large stone), based on their size. Data-dependent and data-independent acquisitions allowed deep profiling of stone proteomics. The proteomic signatures and pathways illustrated major changes as the stones grew. Stones initiate from a small nidus, grow outward, and show major enrichment in ribosomal proteins and factors related to coagulation and platelet degranulation, suggesting a major dysregulation in specific pathways that can be targeted for new therapeutic options.

## Introduction

Cystinuria is a genetic disorder characterized by aggressive/recurrent kidney stone formation. It is caused by mutations in the solute carrier family 3 member 1 (*SLC3A1)*, solute carrier family 7 member 9 (*SLC7A9)*, and/or in the recently identified *AGT1* gene that codes for a cystine reabsorption transporter [1–3]. Patients with cystinuria typically excrete markedly elevated levels of quantitative urinary cystine and develop cystinuric stones at a high rate of recurrence due to the low solubility of cystine. Current interventions aim to decrease urinary cystine concentration with a combination of the following: i) increased fluid intake, ii) a low protein diet, and iii) urine alkalinizing drugs or cystine-binding thiol drugs, such as penicillamine and tiopronin [4]. In spite of these interventions, cystinuric patients experience high stone recurrence rates and endure repeated surgical interventions. Furthermore, these medications can be associated with serious adverse side effects, including decreased renal function [5].

Little is known about the factors that contribute to the severity and recurrence rates of cystinuria-related stone events [6–8]. While some cystinuric patients experience chronic stone formation with multiple surgeries per year, others have few or no stone events throughout their lifetime; this is frequently independent of their quantitative urinary cystine levels. Even when cystinuric patients present above a urine threshold of 300 mg/L cystine output/day, there is typically no correlation between cystine excretion and recurrence rate of stone formation [9]. Cystine output clinically correlates with the ability to reabsorb cystines, and increased cystine output is a risk factor for stone development. Pharmaceutical interventions that target urinary cystine output have had limited efficacy on stone recurrence. These observations suggest that more factors contribute to cystine stone development.

There are only few reports on urinary stone proteomics, but the human urine proteome is well characterized, and large repositories are provided by the Human Kidney and Urine Proteome Project (http://www.hkupp.org/). Human urine proteomics is a promising approach for biomarker discovery, particularly for studying the pathogenesis of kidney diseases and diseases of the urothelial tract [10]. Additionally, proteomic studies of mouse urine show similar protein signatures when age-matched with humans [11]. Cystinuric models are not as well understood, and proteomic studies for patients with cystinuria are lacking. Some studies have been conducted, mostly in children [12]; however, they examined urine rather than the corresponding stones, leaving the stone protein compositions unknown [13].

Urinary proteins are generally believed to contribute to the development of urinary stones by promoting crystal aggregation and adherence to the renal epithelium; additionally, the role and interactions with non-proteinaceous macromolecules, such as calcium oxalate monohydrate (COM) and other components cannot be understated [14, 15]. Reinstatler et al. reported that stones with non-cystine components develop in about ~30% of human patients with cystinuria (as reviewed from records of a multi-institutional cohort of 125 patients with cystinuria), underscoring the importance of continued stone analysis [16].

Matrix proteins have been detected in proteomic profiles of human urinary stones, but due to radiation concerns and poor resolution with clinically available CT imaging, especially in comparison to micro-CT utilized in rodent animal models, scarce information is available on the early events that lead to urinary stone development and the progression of kidney stones. Thus, analysis has been limited to larger, mature stones that have been surgically extracted from patients presenting with acute renal colic.

Using a cystinuric mouse model, a dynamic study on cystinuric stone formation beginning with the smallest aggregates or “sand” to the largest stones categorizing into three other separate sizes was undertaken. For accurate quantification of relative protein abundance, we used label-free proteomic data-independent acquisitions (DIA) or SWATH assays [17, 18] that allow us to accurately determine changes in relative protein expression levels between multiple different sample sets, specifically comparing protein profiles between the different stone sizes and categories. This comprehensive DIA technology provides high sensitivity to quantify changes in relative protein abundance during stone formation. This report highlights the complex organic composition of bladder stones with a constantly changing proteome as stones increase in size. We provide molecular insight into the initiation and growth of these stones, as well as valuable comparisons to human urine composition and kidney disorders.

## Materials and methods

### Reagents and standards

HPLC solvents, or more specifically, high quality LC-MS grade purity solvents (e.g., acetonitrile and water) were obtained from Burdick & Jackson (Muskegon, MI). Reagents for protein chemistry (e.g., iodoacetamide, dithiothreitol (DTT), ammonium bicarbonate, formic acid, and urea) were purchased from Sigma Aldrich (St. Louis, MO). Proteomics grade trypsin was from Promega (Madison WI). HLB Oasis SPE cartridges were purchased from Waters (Milford, MA).

### Mice and micro-computed tomography

All procedures and protocols were approved by the Institutional Animal Care and Use Committee of the Buck Institute for Research on Aging. Male *Slc3a1*^*-/-*^ mice (6–12 weeks old) were anesthetized with isoflurane and scanned using Skyscan 1176 μCT scanner (Bruker Corp, Billerica, MA). The Skyscan reconstruction program NRecon was used for image reconstruction, and bladder stone volume was quantified using the Bruker CT-Analyzer (CTAn, Version 1.14) program with Hounsfield units. 3-D image models were created using CTAn and Bruker CT-Volume (CTVol, Version 2.2).

### Stone collection and sample preparation

Batches of cystine sand and stone samples were harvested from the bladder of male *Slc3a1*^*-/-*^ mice in at least four biological replicates. Sand and stone samples were categorized, according to their aggregate surface characteristics and apparent diameters. Sand samples were granular and small in diameter (<1 mm^2^), and stone samples were lithic and larger in diameter (>5 mm^2^). All sand and stone samples were washed thoroughly in deionized water to remove contaminants and debris. Dried samples were then ground into a powder in a mortar and pestle. To extract proteins from the sand and stone samples, we used a modified version of the protocol of Jiang *et al*. [19]. Briefly, ground stone and sand samples were lysed in 6 M guanidine HCl, 100 mM Tris (pH 7.4), and Sigma-Aldrich complete EDTA-free protease inhibitor cocktail. The samples were incubated at 4°C for 72 hours, and the supernatant was collected.

### Cystine determination of stones

Cystine sand and stone samples that were harvested from the bladder of male *Slc3a1*^*-/-*^ mice were washed thoroughly in deionized water. Dry samples were then ground into a powder in a mortar and pestle. Samples were sonicated and solubilized in water at 37°C for 16 hours with shaking, and the supernatant was collected for analysis.

### Sample processing for mass spectrometry

The protein mixture (typically 100 μg protein lysate) was reduced with 20 mM DTT (37°C for 1 hour), and then alkylated with 40 mM iodoacetamide (30 min at RT in the dark). Samples were diluted 10-fold with 100 mM Tris, pH 8.0, and incubated overnight at 37°C with sequencing grade trypsin (Promega) added at a 1:50 enzyme:substrate ratio (wt/wt). Samples were then acidified with formic acid and desalted using HLB Oasis SPE cartridges (Waters). Proteolytic peptides were eluted, concentrated to near dryness by vacuum centrifugation, re-suspended and further desalted (C-18 zip-tips) for insoluble protein mass spectrometric analysis.

### Mass spectrometry acquisition and analysis

Samples were analyzed by reverse-phase HPLC-ESI-MS/MS with an Eksigent Ultra Plus nano-LC 2D HPLC system (Dublin, CA), combined with a cHiPLC System, and directly connected to a quadrupole time-of-flight TripleTOF 6600 (QqTOF) mass spectrometer (SCIEX). Briefly, DIA acquisitions acquire MS/MS fragment ions from essentially all peptide precursor ions by consecutively passing large *m/z* DIA segments over the MS1 scan range and acquiring high-resolution MS/MS scans for each Δm/z segment. Typically, we scan from *m/z* 400–1250 with 64 variable window m/z segments [20–22].

Typically, mass resolution for MS1 scans and corresponding precursor ions was ~45,000, and resolution for MS/MS scans and resulting fragment ions was ~15,000 (‘high-sensitivity’ product ion scan mode). For acquisition, the autosampler was operated in full injection mode overfilling a 3-μL loop with 4 μL of analyte for optimal sample delivery reproducibility. Briefly, after injection, peptide mixtures were transferred onto a trap chip (with 200 μm x 6 mm ChromXP C18-CL chip, 3 μm, 300 Å, SCIEX) and washed at 2 μL/min for 10 min with the loading solvent (H_2_O/0.1% formic acid). Subsequently, peptides were transferred to each 75 μm x 15 cm ChromXP C18-CL chip, 3 μm, 300 Å, (SCIEX), and eluted at a flow rate of 300 nL/min with the following gradient: at 5% solvent B in A (from 0–5 min), 5–8% solvent B in A (from 5–12 min), 8–35% solvent B in A (from 12–67 min), 35–80% solvent B in A (from 67–77 min), at 80% solvent B in A (from 77–87 min), with a total runtime of 120 min, including mobile phase equilibration. Solvents were prepared as follows, mobile phase A: 2% acetonitrile/98% of 0.1% formic acid (v/v) in water, and mobile phase B: 98% acetonitrile/2% of 0.1% formic acid (v/v) in water.

Data-dependent acquisitions (DDA) were performed on the TripleTOF 6600 to obtain MS/MS spectra for the 30 most abundant precursor ions (100 msec per MS/MS) after each survey MS1 scan (250 msec), yielding a total cycle time of 3.3 sec as described [23, 24]. For collision-induced dissociation tandem mass spectrometry (CID-MS/MS), the mass window for precursor ion selection of the quadrupole mass analyzer was set to ± 1 m/z. Each of the 21 stone samples was acquired in two technical replicates (MS injection replicates) using the Analyst 1.7 (build 96) software. All database search results and details for peptide identifications are provided in **S1 Table.** For additional quantitative assessments data-independent acquisitions (DIA), or SWATH acquisitions, were performed for four ‘sand’ stone samples and four large stone samples, plus additional small (2x) and medium (2x) stone samples. Briefly, instead of the Q1 quadrupole transmitting a narrow mass range through to the collision cell, a wider window of variable window width (5–90 *m/z*) is passed in incremental steps over the full mass range (*m/z* 400–1250 with 64 DIA segments, 45 msec accumulation time each, yielding a cycle time of 3.2 sec that includes one MS1 scan with 250 msec accumulation time). The variable window width is adjusted, according to the complexity of the typical MS1 ion current observed within a certain *m/z* range using a SCIEX ‘variable window calculator’ algorithm (more narrow windows were chosen in ‘busy’ *m/z* ranges, wide windows in *m/z* ranges with few eluting precursor ions). DIA workflows produce complex MS/MS spectra, which are a composite of all the analytes within each selected Q1 *m/z* window.

### Mass spectrometry database search

Mass spectrometric data was searched using the database search engine Protein Pilot [25] (SCIEX 5.0, revision 4769) with the Paragon algorithm (5.0.0.0.4767). The search parameters were set as follows: trypsin digestion, cysteine alkylation set to iodoacetamide, and *Mus musculus* as species (33,338 protein entries, SwissProt database release 2014_05). Additional searches utilized phosphorylation emphasis. Trypsin specificity was assumed as C-terminal cleavage at lysine and arginine. Processing parameters were set to "Biological modification" and a thorough ID search effort was used. For database searches, a cut-off peptide confidence value of 99 was chosen, and a minimum of two identified peptides per protein was required. The Protein Pilot False Discovery Rate (FDR) analysis tool, and the Proteomics System Performance Evaluation Pipeline (PSPEP) algorithm provided a global FDR of 1% and a local FDR at 1% in all cases.

### Mass spectrometry data processing and quantification

#### Quantitative processing of DIA data

DIA data was processed with Spectronaut Pulsar v12 (12.020491.3.1543) software [26] from Biognosys, using a spectral ion library generated from the data-dependent acquisitions. In addition, we used Skyline 3.5 [27], an open source software project (http://proteome.gs.washington.edu/software/skyline), to process DIA data for relative quantitation comparing large stone and sand samples. For the DIA MS2 data sets, Skyline quantitation was based on XICs of up to 10 MS/MS fragment ions, typically y- and b-ions, matching to specific peptides in the spectral libraries used. Significance was assessed using q-values from two-tailed t-tests adjusted for multiple testing corrections. Significantly changed proteins were accepted at a 5% FDR (q-value < 0.05). All database search results and details for peptide identifications and quantitation are provided in **S2 Table.** Pathway enrichments were determined with the ConsensusPathDB tool (http://cpdb.molgen.mpg.de). Heatmaps were generated in R using the heatmap.2 function contained within the gplots package. The Ward method was used for clustering of heatmaps.

#### Raw data accession

The mass spectrometric raw data associated with this manuscript may be downloaded from MassiVE at ftp://massive.ucsd.edu/MSV000087116/

MassIVE ID number: MSV000087116; ProteomeXchange number: PXD025036.

## Results

### Cystine stone development is characterized by a sediment-like precursor, a stone nidus, and growth from the nidus

**Fig 1:**
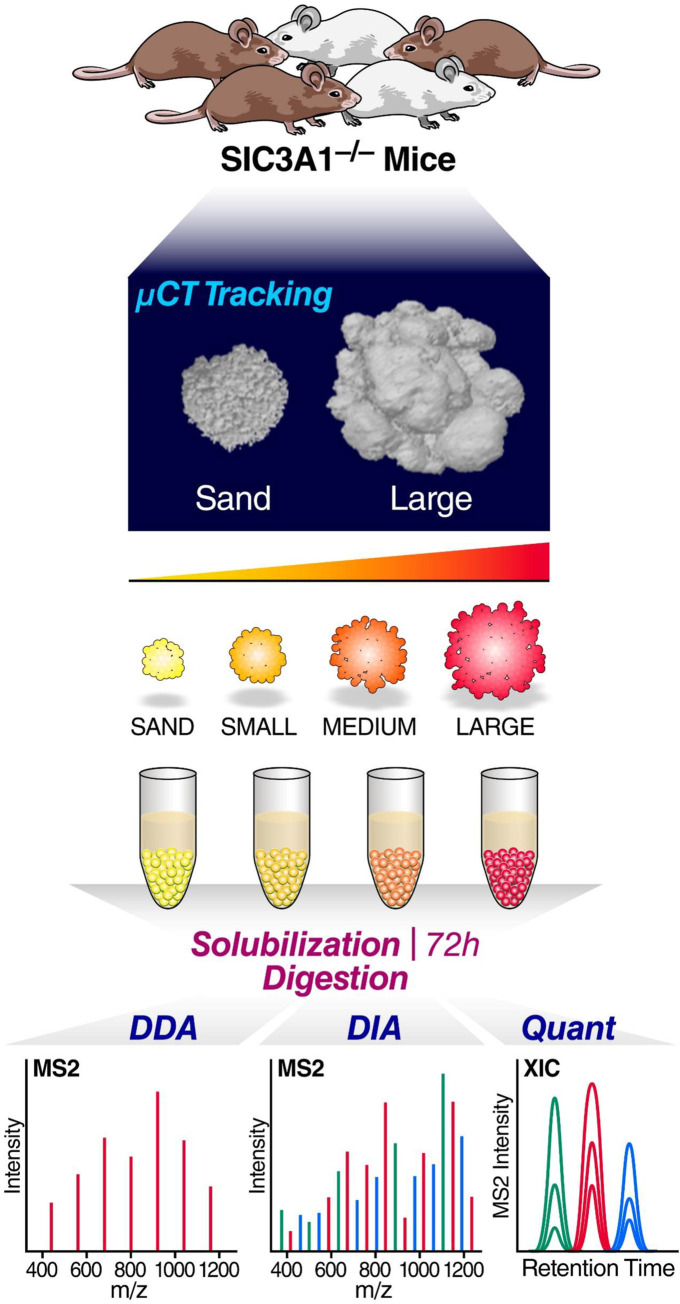
Workflow for tracking and proteomic analysis of *Slc3a1-/-* mouse cystinuric stones. Shows representative images for the tracking of mouse stones using μCT, collecting and solubilizing for mass spectrometry analysis using both DDA and DIA.

Mice deficient for either the *Slc3a1* or *Slc7a9* gene develop cystine urinary stones, corresponding to similar stone types in human cystinuria [28]. We utilized these mouse models to study the initiation and progression of these cystinuric stones **(Fig 1)**. Unlike most urinary human cystine stones, mouse urinary stones manifest primarily in the bladder. This feature of murine stone formation enabled *in vivo* imaging of urinary stone development in an environment without the physical constraints of the renal medulla. The early stages of stone formation are thus clearly discernable by μCT. To briefly describe our overall workflow as shown in Fig 1, we initially image the sand-like material, and various sizes of stones by i) in vivo μCT scanning, ii) harvesting of the ‘sand’ and small, medium and large stones, followed by solubilization and proteolytic digestion, iii) mass spectrometric discovery of proteins that are present in the sand and stones using data-dependent acquisition (DDA) for deep library building, and iv) finally acquiring data-independent acquisitions (DIA) for all samples for comprehensive quantification of the proteins that are changing, comparing proteins isolated from sand-like material with proteins obtained from the various other stone sizes, specifically focusing on the dynamic changes between sand and large stones.

**Fig 2:**
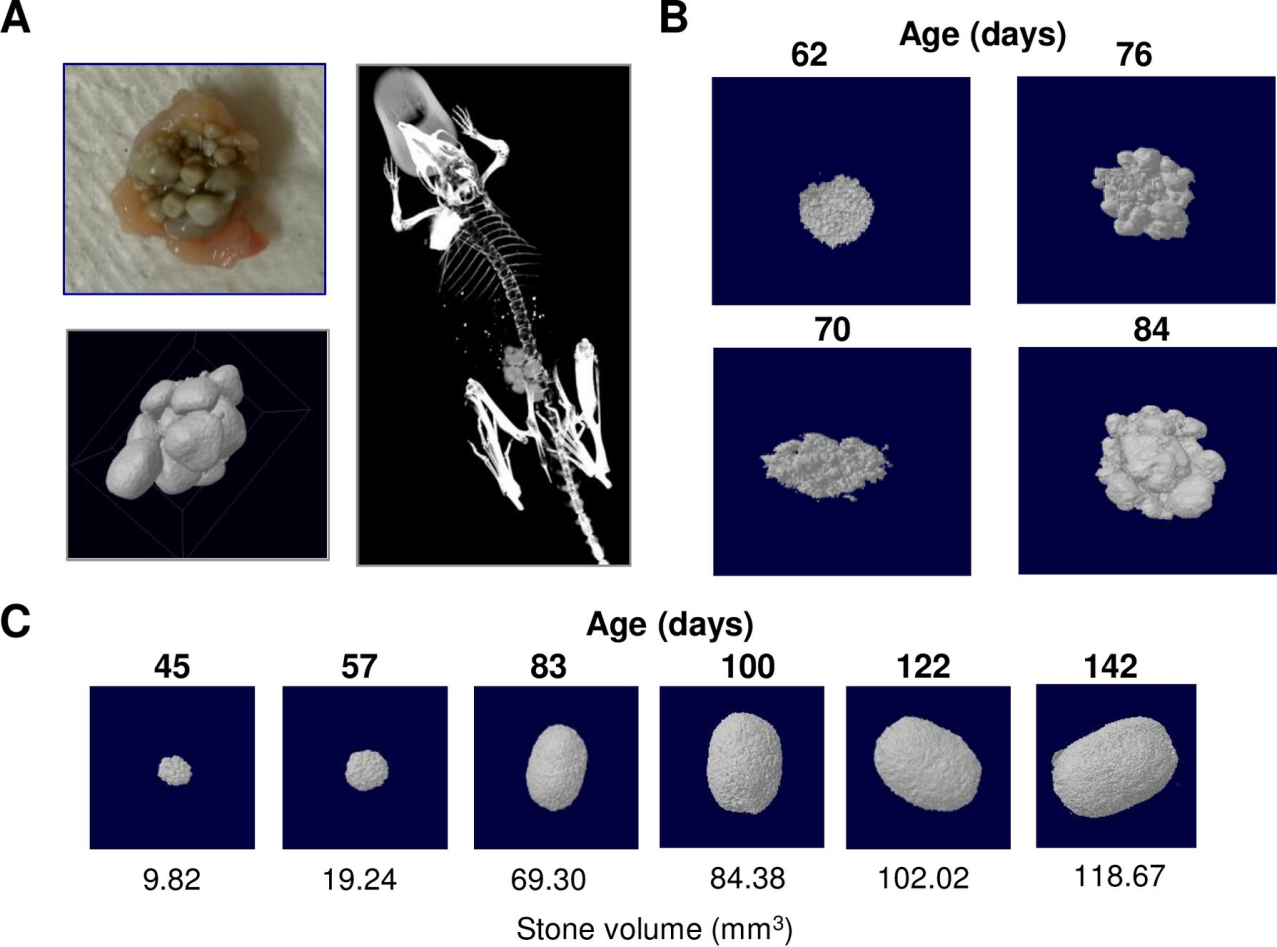
Stages of stone development. (A) μCT imaging and 3D modeling of *in vivo* cystine stone development in a representative *Slc3a1*^*-/-*^ mouse. (B) μCT imaging and 3D modeling of urinary stone growth in an individual *Slc3a1*^*-/-*^ mouse. Stone formation often initiates as sediment in the bladder (P62), and progresses through agglomeration (P70–76) to form urinary stones (P84); this progression can be seen here. (C) μCT imaging and 3D modeling of a single stone’s growth in mm^3^ over 142 days.

Using μCT to examine individual mice, we found that stone formation is initiated by urinary cystine sediment that accumulates in the bladder (**Fig 2A**). In 15 of 16 mice analyzed, a sediment-like stage was detected prior to the presence of stones, subsequently referred to as “sand”. The sediment was characterized by its granular size and shape in Hounsfield units that correspond to the radiodensity of cystine stones. These observations suggest that the sediment is a precursor of stones in mice and that the sediment aggregates in a remodeling process to form distinct stones. According to the μCT images of multiple animals, a "sand" stage was observed in the bladder that preceded before most stone formation.

In *Slc3a1*^*-/-*^ mice, stones of different sizes typically develop in parallel, however, the accumulation of sand precedes and initiates the stone formation, and this was visualized by the μCT tracking. It appears that individual sand particles begin accumulating before their eventual, continuous growth into fully developed stones. The individual sand particles will continue to form, and subsequently develop into stones, and grow alongside with other particles and stones of various sizes at the same time, leading to many stones of different sizes growing at the same time. We analyzed the growth of individual stones that were discernable by their distinct sizes. We found that cystine stones initiate as individual nidi and accumulate volume in a gradual, appositional process (**Fig 2B and 2C**). From here forward, the stones will be referred to as “sand”, “small”, “medium” and “large” stones. From these *in vivo* observations, we propose a model of cystine stone development that depends on accumulation and formation of cystine crystal sediment. The granular sand was categorized and differentiated from the smallest stones both by color but more importantly by texture. The sand particles were more irregularly shaped, rough textured and most of the time yellow in color, whereas the formed (and growing) stones were rounded, smooth and gray in color. In summary, a nidus forms from this yellow, granular precursor and develops into a rounded, smooth urinary stone.

### Cystine stone development is associated with changes in the protein matrix

**Fig 3:**
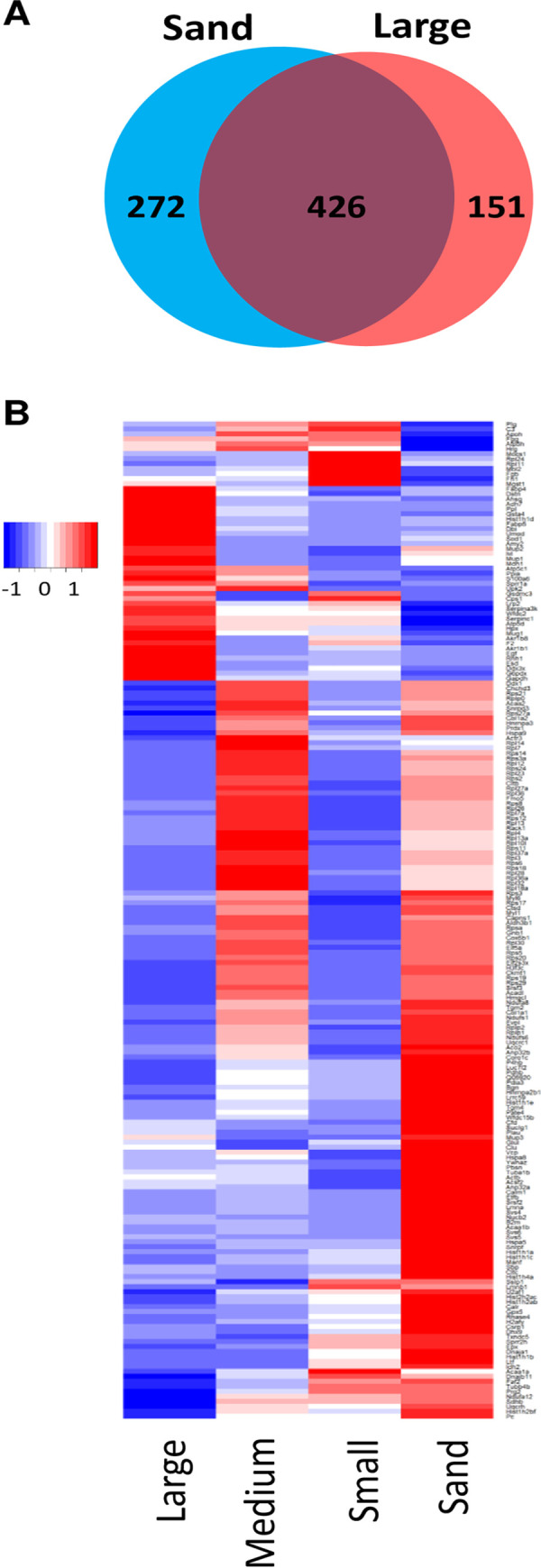
Proteomic analysis of stone development in large and sand kidney stones. (A) Venn diagram comparing the proteins identified in sand-sized stones (698 proteins) versus large stones (577 proteins). We identified 426 proteins in both fractions. (B) Heatmap of all proteins that were significantly changed in abundance in small, medium, and large versus sand stones (q-value < 0.05) by at least 1.5-fold (200 proteins total). Heatmap colors represent the log2 fold-change of each protein versus the median value in the fraction.

To understand the contribution of the protein matrix to cystine stone formation, we used mass spectrometry to characterize the proteome of the different stages of stone formation in the cystinuric mouse: the cystine sediment and large cystine stones. To assess the proteins associated with the stone matrix, we used sample preparation protocols for protein extractions from bone [19]. Overall, we identified 1034 unique proteins from all stone types (**S1 Table**). Interestingly, of the 849 proteins initially detected in the sand and large sizes by DDA acquisition, 426 were found in common in sand and large stones (**Fig 3A**). This suggests that over half of the proteins found in sand are also represented in the largest stones. These results from our DDA profiling were used to build spectral libraries for subsequent quantification (DIA-MS). Using the highly accurate relative quantification from the subsequent proteomic DIA workflow, we determined significant abundance differences in over 400 proteins across the stone proteome when comparing large stones vs sand (significant changes are listed in **S2 Table**). We found a constantly changing organic matrix in these stones, and interestingly, each size distinction displayed its own signature (**Fig 3B**). These results suggest that specific urinary proteins and other biomolecules have roles in cystine stone development.

**Fig 4:**
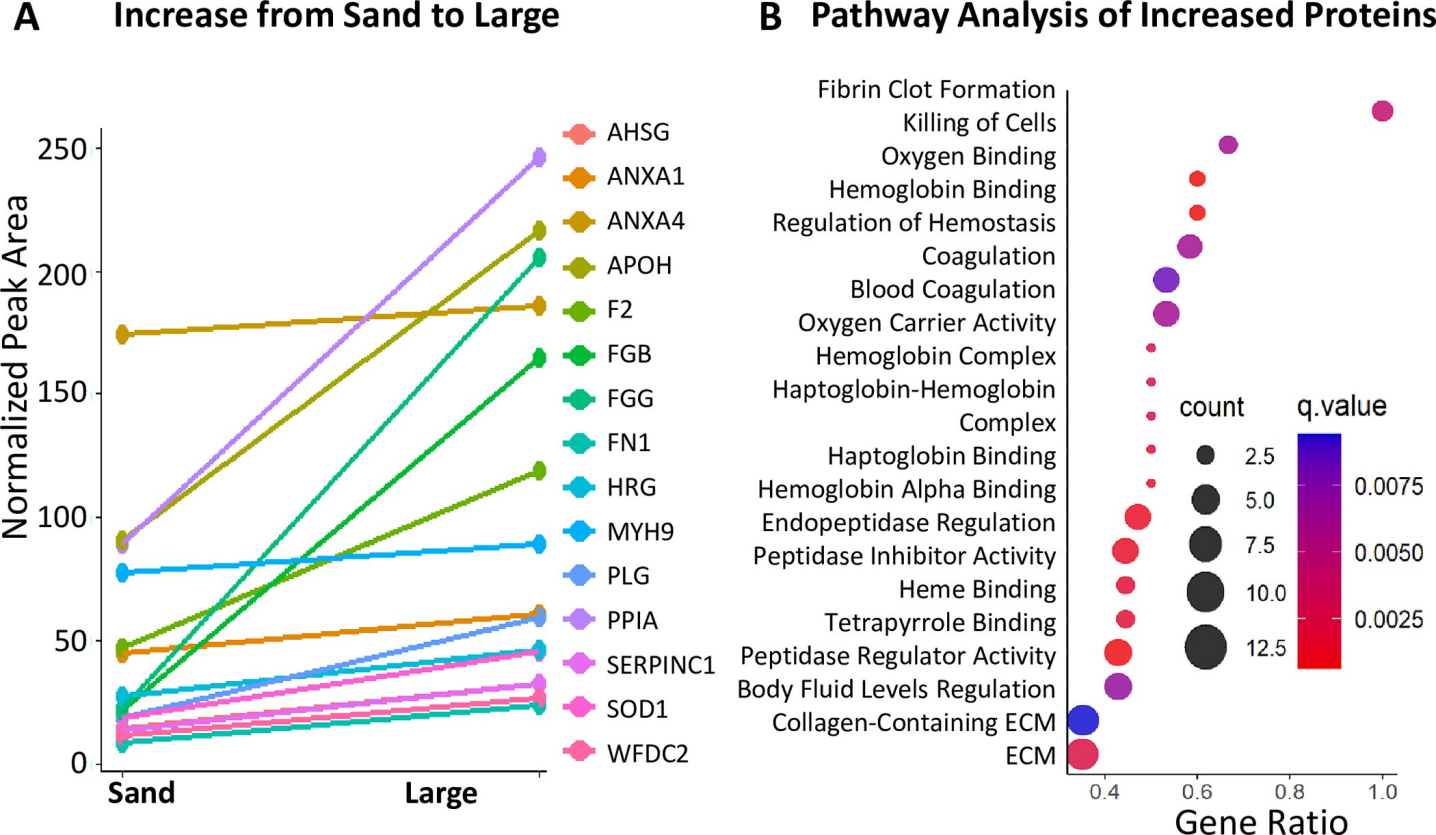
Pathway analysis of significantly changed proteins comparing large stones vs sand. (A) Among upregulated pathways and proteins in large stones (*when comparing large stones vs sand*), coagulation machinery and protease inhibitors are significantly increased. (B) There is also enrichment for coagulation-related pathways and hemoglobin binding and oxygen binding pathways.

**Fig 5:**
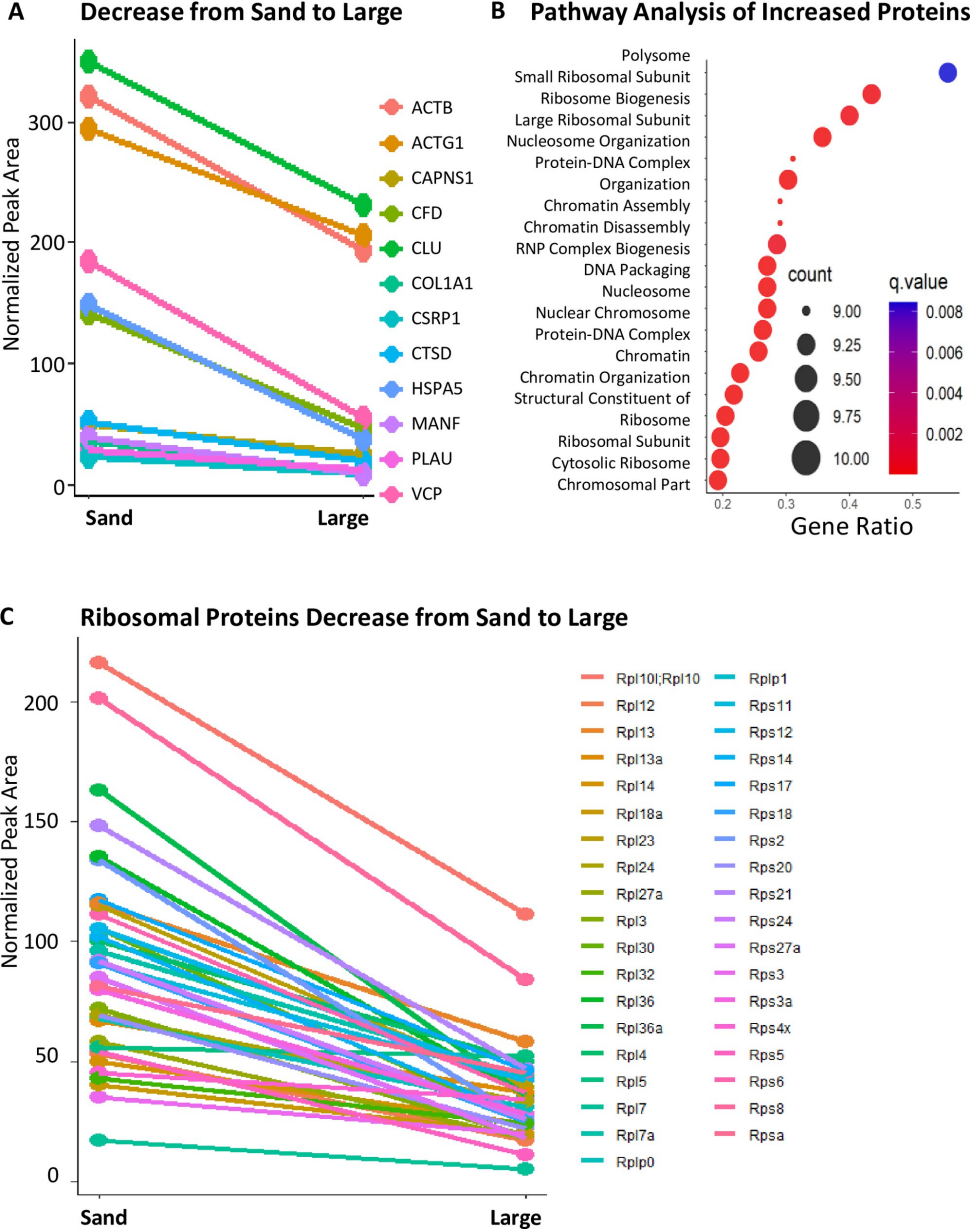
Pathway analysis of significantly changed proteins comparing large stones vs sand. (A) Among downregulated pathways and proteins in large stones (*when comparing large stones vs sand*), RNA export, ribosomal binding and proteases are significantly decreased. (B) Polysome and ribosomal-related pathways are most significantly enriched, but there is also significant enrichment for chromosomal pathways. (C) Levels of many ribosomal proteins decrease drastically as ‘sand’ continues to grow into the large stones.

We then determined what GO pathways were altered by comparing large and sand-type stones. We performed the GO analysis using the significantly changing proteins when comparing sand to large stones (Q<0.05). We show both upregulation and downregulation of multiple pathways, including blood coagulation, ribosomal enrichment, metabolism, and RNA processing and transport (**Figs 4 and 5**). We also compared the identified proteins from the mouse bladder stones with reported proteomes, such as the human urine proteome (Human Proteome Project, HPP). Of the proteins detected in mouse bladder stones, 643 were described as homologous proteins in human urine (Peptide Atlas human urine HUPO [13, 29–33]) (**S3 Table**). In addition, we compared our list of bladder stone proteins with proteins reported from other bladder or kidney stone studies [29–33]. As in humans, these mice show markers of kidney injury, increased fibrinolysis activity, and dysregulation of proteolysis with the change of both proteases and protease inhibitors. These observations suggest that cystinuric mice share reported proteomic profiles obtained from human urine, bladder and kidney studies, which is highly relevant for the potential future applications of this specific mouse model.

### Early stone formation is associated with significant enrichment of ribosomal proteins

Amounts of ribosomal proteins, such as the ribosomal protein RPL13, were much greater in the sand samples than in the large stone samples (**Fig 5C**). Initial seeding of a stone may recruit any of the abundant proteins in the urine [11, 34]. A report of all significantly regulated proteins during stone formation and growth can be found in S1 Table.

### Significant changes in coagulation factors across stone size progression

**Fig 6:**
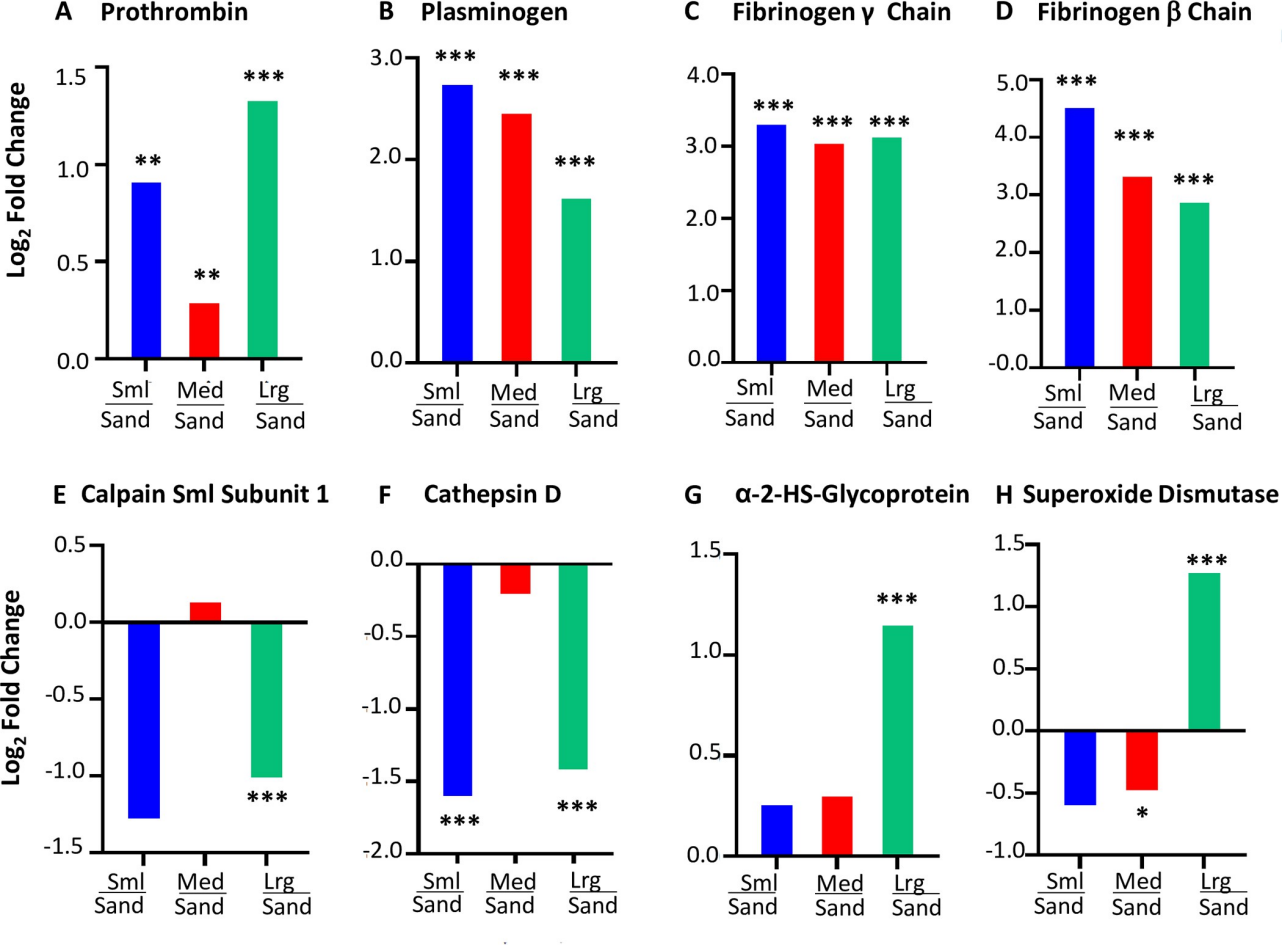
Changes in diverse proteins detected displaying log-scale fold-changes. The bar graphics all display a ratio (stone vs sand), or more specifically: log2-fold changes comparing either ‘small vs sand’, the comparison ‘medium vs sand’, and the comparison ‘large vs sand’ for all shown proteins. (A) Levels of prothrombin increased across all stone sizes (compared to sand), but most drastically when comparing large stones vs sand. (B) Plasminogen (PLG) shows the largest increase in small stones vs sand, and continues to show upregulation in medium/sand and large/sand. (C-D) Fibrinogen gamma-chain (FGG) shows large increases across all stone sizes when compared to sand, and beta chain (FGB) displays the largest increase in small stones vs sand, and continues to show upregulation in medium/sand and large/sand. (E-F) Lower levels of proteases Calpain 4 and Cathepsin D are found in large stones than in sand. (G) Protease inhibitor AHSG was increased in large stones vs sand. (H) SOD1 is most drastically changed in the large stones, compared to sand. (* = p<0.05; *** = p<0.0005).

Using our understanding of stone formation, we determined how the stones changed as they increased in size. We found that their proteomic signatures changed drastically as stones grew from sand to large-sized stones (**Fig 3**). These distinct sizes allowed us to elucidate where the most drastic proteomic changes occurred. In particular, we used the two extremes of large-sized stones and sand to form comparisons in the following analyses. For example, we found a downregulation in the urokinase responsible for activating plasminogen (PLAU) (**Fig 5A**). Similarly, the relative abundance of protease proteins was greater in sand than in large stones, as shown in the downregulated network (**Fig 5A**). We identified a small, but statistically significant, upregulation of prothrombin (F2), another protein involved in coagulation, across all stone sizes, when compared to the smallest sand size (**Fig 6A**). Fibrinogen side chains (e.g., FGB and FGG) and plasminogen (PLG) were significantly more prevalent across all sizes in comparison to sand (**Fig 6B–6D**). These changes in the stone protein profiles possibly reflect and indicate major alterations in the clotting pathway as part of disease progression.

### Protease and protease inhibitor activities are indicative of stone growth

Proteases and protease inhibitors appear most drastically altered in the larger categories. Levels of cysteine protease Calpain 4 and the aspartyl protease Cathepsin D were significantly lower in small stones compared to sand, and they were significantly lower in large stones compared to sand, the value of the differential downregulation was similar around log2-fold = -1.5 for both small/sand as well as large/sand (**Fig 6E and 6F**). Alternatively, more protease inhibitor alpha-2-HS-glycoprotein (AHSG) was found in the large stones than in the sand (**Fig 6G**). Using this trend, we hypothesize that restriction of protease activity may also have a role in stone formation or expansion. Interestingly, SOD1 was increasingly more prevalent as the stones grew (**Fig 6H**). SOD1 activity is required for processing superoxide, and the effect of its elevated presence in only the largest stones is rather interesting.

## Discussion

To gain insight into the cystine stone development process, we applied longitudinal μCT scanning to characterize early stone formation and initiation in a mouse model of cystinuria (*Slc3a1*^*-/-*^*)*. These mice lack the transporter required to reabsorb cystine and develop cystinuric stones throughout their life. We then used in-depth proteomic analysis to identify the protein changes associated with the different stages of stone formation and growth. By combining imaging and proteomic analysis of these stages of cystine stone formation, we found stone matrix protein changes that are associated with cystine stone maturation. These formation-associated stone proteins may provide information on stone formation mechanisms and the systems that promote stone development including growth and maturation. We also recapitulated and significantly expanded on the existing data [35, 36], including observations of upregulation of fibrosis and inflammation factors, but we have also captured interesting distinctions in the varying sizes of collected stones that would have not been possible using humans as the primary model. Because current treatments of cystinuria are not effective in the long term, it is necessary to discover new therapeutic targets to treat this disorder.

Many of the bladder stone proteins we identified in this study are also found in human calcium-based stones and in the human urine proteome [11, 34, 37]. Proteins that are overrepresented in the early development of the cystine stones (*sand*) include ribosomal proteins and metabolic factors in the electron transport chain and TCA cycle. In large stones, the relative increased levels of coagulation factors, protease inhibitors, and SOD1 suggest that injury and inflammation may be a normal part of stone formation. Hydrogen peroxide also reacts with free cysteine to form the oxidized dimer cystine, reportedly leading to kidney stone formation [38]. Interestingly, we found SOD1, which generates H_2_O_2_ from superoxide, at higher relative abundances in large stones. Accumulation of SOD1 within the stones may promote local conditions that include a higher local H_2_O_2_ and lower pH that contributes to stone development. All of these factors need to be investigated further in reference to stone initiation and expansion. However, this is a first study showing how complex and dynamic the protein profiles are inside growing cystine stones. Also, the initial nidus of the urinary stone may cause a level of inflammation that signals for the coagulation response and is bolstered by a lack of relevant proteases and an increase in protease inhibitors to clear constantly amalgamating protein clumps. As a consequence, the stones are never properly dissolved in the body and continue to grow into larger stones. In this study, there were significant increases in protease inhibitors, such as the Serpin family of protease inhibitors, and significant decreases in cysteine proteases, CAPN1 and 4, as well as the aspartyl protease Cathepsin D. Therefore, these proteases cannot clear amalgamating peptides, which could lead to decreased stone solubility in susceptible bladders. Interestingly, patients treated for HIV with Atazanavir have an increased risk of developing stones [39, 40]. Atazanavir is a protease inhibitor that specifically inhibits HIV-1 protease, an aspartyl protease like the Cathepsin proteases. More experiments are needed to identify a method of stone initiation involving these molecules, but these data suggest a link between a lack of protease activity and an increase in stone formation [39, 41]. Our analysis also identified multiple important clotting factors as significantly upregulated in large stones.

The protease inhibitor alpha-2-hs-glycoprotein (AHSG) is also interesting. We found that its presence was increased significantly only in the large stones and was equally found in small and medium, compared to sand. This glycoprotein is upregulated in urine studies linking its expression to an increased risk for kidney disease [42]. AHSG knockdown in mouse models lead to calcification of the vasculature [43]. These mouse models are relatively well studied, but more kidney research is needed to determine any specific role of AHSG in the progression of these disorders. Because the knockout models lead to calcification, AHSG could be involved in a protective molecular mechanism in response to kidney injury. Our data suggest that, as the stones increase in size from medium to large, AHSG accumulates past the levels seen in the other size distinctions. Its role in calcification and known status as a circulating protein make it an important biomarker for multiple kidney- and bladder-related disorders.

Clotting activity is complex and highly regulated through different protein pathways. In this study, we detected significant fold-changes, both increasing and decreasing, across all stone sizes. Some of these factors include components that make up blood clots, fibrinogen side chains, and the machinery regulating their degradation, such as plasmin and antithrombin. Two of the known fibrin subunits (FGG and FGB) were upregulated across all stone sizes. Indeed, patients with chronic kidney disease are more likely to have blood clot clearance issues and show a proteomic signature similar to these stones [35, 36].

We determined that levels of PLG were higher in all stones than in sand stones. Interestingly, PLG showed similar fold-change patterns across stone sizes when compared to fibrin beta chain, which was most drastically increased in the small stones. Increases in the fibrin side-chains tapered off as the stones get larger. PLG, while responsible for degradation of the fibrin subunits, must be cleaved to be activated. This suggests that, even upon an increase in PLG expression, a large amount of fibrin clots could be left intact. This led us to examine the levels of one of the proteins responsible for the cleavage of the inactive plasminogen, urokinase-type plasminogen activator (PLAU). PLAU levels were decreased across all stone sizes, specifically and significantly in the larger stones. However, levels of prothrombin, another crucial component of the clotting machinery, were significantly higher in stones than sand samples. Thrombin is responsible for the formation of the insoluble fibrin clots by mediating the cleavage of fibrinogen to fibrin. Prothrombin as found across all stone sizes, suggesting and reflecting active clotting processes in the disease model. Other stone-associated factors may contribute to the stone matrix proteome.

The significance of these differences between the cystine sediment and sizable formed stones remains to be defined. Our analyses of mouse cystine stone formation reveal that these cystine stones contain a significant organic matrix component, which has not been reported to date. We determined that formation of large stones is preceded by deposition of urinary cystine ‘sand’-like particles, which are likely precursors of the growing stones. Interestingly, we believe that the protein changes in the urine of these cystinuric mice are reflected in the protein profiles that we discovered in the various bladder stones. We do think it is likely that proteins from the urine bind to cystine crystals as the aggregates increase in size during this dynamic process. Future experiments could be performed that directly link the urine proteome to the protein compositions in the bladder stones; we have shown the large overlap of known urine proteins (HKUPP) with the bladder stone components. By combining imaging analysis and proteomic analysis, we discovered a stone proteome that is constantly changing as stones develop, and we also propose possible mechanisms by which stone formation and growth occur and that could be explored in future studies. These findings could be useful when typical methods of treatment still lead to stone reoccurrence. Here we provide druggable targets in coagulation machinery, protease activity, as well as some metabolic proteins. It is also important to consider stone formation when administering protease inhibitors, such as Atazanavir, as these drugs may impede cystine reabsorption or otherwise contribute to stone formation.

## Supporting information

S1 Table Identification details from DDA acquisitions of cystinuric stones. **A)** Identified proteins from DDA acquisitions of cystinuric stones, all samples combined. **B)** Identified proteins from DDA acquisitions bladder stones, all samples combine. **C)** Identified cystinuric stone proteins with peptide ID details from DDA acquisitions, all samples combined.
(XLSX)

S2 Table DIA Quantification–significantly changing proteins. **A)** Significantly changing proteins found when comparing large stones vs sand. **B)** All significantly changing proteins found when comparing large stones vs sand. **C)** Significantly changing proteins found when comparing medium stones vs sand. **D)** Significantly changing proteins found when comparing small stones vs sand. **E)** Significantly changing proteins found across all comparisons.
(XLSX)

S3 Table Human kidney urine proteome project- Shared proteins between mouse cystinuric stones and the HKUPP database. (XLSX)

S4 Table Supplemental Table for Fig 3B: Heatmap across stone sizes. (XLSX)

S5 Table Increasing proteins in stones compared to sand. **A)** Supplemental Table for Fig 4A: Coagulation and Fibrinolysis Proteins That Increased Compared to Sand. **B)** Supplemental Table for Fig 4B: Gene Ontology Analysis for Increased Proteins. **C)** Supplemental Table for Fig 4B: Gene Ontology Output.
(XLSX)

S6 Table Decreasing proteins in stones compared to sand. **A)** Coagulation and Fibrinolysis Proteins That Decreased Compared to Sand. **B)** Gene Ontology Analysis. **C)** Gene Ontology Output. **D)** Ribosomal Protein Line Plot.
(XLSX)

## Abbreviations

MS1: full scan mass spectrum
MS/MS: tandem mass spectrometry
DTT: dithiothreitol
XIC: extracted ion chromatogram
DDA: data-dependent acquisition
DIA: data-independent acquisition
FDR: false discovery rate
CV: coefficient of variation
